# Supplementary material for: Design and Synthesis of New Coumarin Hybrids Active Against Drug-Sensitive and Drug-Resistant Neuroblastoma Cells
Source: Antioxidants (Basel). 2025 Dec 24;15(1):31. doi: 10.3390/antiox15010031 (PMC12837142; doi:10.3390/antiox15010031)

Supporting Materials

Design and synthesis of new coumarin hybrids targeting drug sensitive and resistant neuroblastoma cells

Carola Grondona [1], Barbara Marengo [2,3]; Giulia Elda Valenti [2,]; Eleonora Russo [1]; Cinzia Domenicotti [2,3*] and Bruno Tasso [1*]

[1] Department of Pharmacy, University of Genoa, Viale Benedetto XV 3, I-16132 Genoa, Italy
[2] Department of Experimental Medicine, University of Genoa, Via Leon Battista Alberti 2, I-16132 Genoa, Italy
[3] IRCCS Ospedale Policlinico San Martino, Genoa, Italy
* Correspondence: cinzia.domenicotti@unige.it; Bruno.Tasso@unige.it

Summary

– **Figure S1-S2.** ^{1}H and ^{13}C NMR spectra of *(E)-N′-(3-methoxy-4-phenoxybenzylidene)-2-oxo-2H-chromene-3-carbohydrazide* **1.** ………… S1
– **Figure S3-S4.** ^{1}H and ^{13}C NMR spectra of *(E)-N′-(4-(benzyloxy)-3-methoxybenzylidene)-2-oxo-2H-chromene-3-carbohydrazide* **2.** ………… S2
– **Figure S5-S6.** ^{1}H and ^{13}C NMR spectra of *(E)-N′-(4-((4-fluorobenzyl)oxy)-3-methoxybenzylidene)-2-oxo-2H-chromene-3-carbohydrazide* **3**. ………… S3
– **Figure S7-S8.** ^{1}H and ^{13}C NMR spectra of *(E)-N′-(4-methoxy-3-phenoxybenzylidene)-2-oxo-2H-chromene-3-carbohydrazide* **4**. ………… S4
– **Figure S9-S10.** ^{1}H and ^{13}C NMR spectra of *(E)-N′-(3-(benzyloxy)-4-methoxybenzylidene)-2-oxo-2H-chromene-3-carbohydrazide* **5**. ………… S5
– **Figure S11-S12.** ^{1}H and ^{13}C NMR spectra of *(E)-N′-(3-((4-fluorobenzyl)oxy)-4-methoxybenzylidene)-2-oxo-2H-chromene-3-carbohydrazide* **6**. ………… S6
– **Figure S13-S14.** ^{1}H and ^{13}C NMR spectra of *(E)-8-methoxy-N′-(3-methoxy-4-phenoxybenzylidene)-2-oxo-2H-chromene-3-carbohydrazide* **7**. ………… S7
– **Figure S15-S16.** ^{1}H and ^{13}C NMR spectra of *(E)-N′-(4-(benzyloxy)-3-methoxybenzylidene)-8-methoxy-2-oxo-2H-chromene-3-carbohydrazide* **8**. ………… S8
– **Figure S17-S18.** ^{1}H and ^{13}C NMR spectra of *(E)-N′-(4-((4-fluorobenzyl)oxy)-3-methoxybenzylidene)-8-methoxy-2-oxo-2H-chromene-3-carbohydrazide* **9**. ………… S9
– **Figure S19-S20.** ^{1}H and ^{13}C NMR spectra of *(E)-8-methoxy-N′-(4-methoxy-3-phenoxybenzylidene)-2-oxo-2H-chromene-3-carbohydrazide* **10**. ………… S10
– **Figure S21-S22.** ^{1}H and ^{13}C NMR spectra of *(E)-N′-(3-(benzyloxy)-4-methoxybenzylidene)-8-methoxy-2-oxo-2H-chromene-3-carbohydrazide* **11**. ………… S11
– **Figure S23-S24.** ^{1}H and ^{13}C NMR spectra of *(E)-N′-(3-((4-fluorobenzyl)oxy)-4-methoxybenzylidene)-8-methoxy-2-oxo-2H-chromene-3-carbohydrazide* **12**. ………… S12
– **Figure S25-S26.** ^{1}H and ^{13}C NMR spectra of *(E)-8-hydroxy-N′-(3-methoxy-4-phenoxybenzylidene)-2-oxo-2H-chromene-3-carbohydrazide* **13**. ………… S13
– **Figure S27-S28.** ^{1}H and ^{13}C NMR spectra of *(E)-8-hydroxy-N′-(4-methoxy-3-phenoxybenzylidene)-2-oxo-2H-chromene-3-carbohydrazide* **14**. ………… S14
– **Figure S29-S30.** ^{1}H and ^{13}C NMR spectra of *(E)-N′-(3-(benzyloxy)-4-methoxybenzylidene)-8-hydroxy-2-oxo-2H-chromene-3-carbohydrazide* **15**………… S15
– **Figure S31-S32.** ^{1}H and ^{13}C NMR spectra of *(E)-N′-(3-((4-fluorobenzyl)oxy)-4-methoxybenzylidene)-8-hydroxy-2-oxo-2H-chromene-3-carbohydrazide* **15**. ………… S16
– **Figure S33-S34.** ^{1}H and ^{13}C NMR spectra of *(E)-N′-(3,4-dihydroxybenzylidene)-8-hydroxy-2-oxo-2H-chromene-3-carbohydrazide* **17**. ………… S17

Figure S1. ^{1}H NMR spectra of *(E)-N′-(3-methoxy-4-phenoxybenzylidene)-2-oxo-2H-chromene-3-carbohydrazide* (**1**).

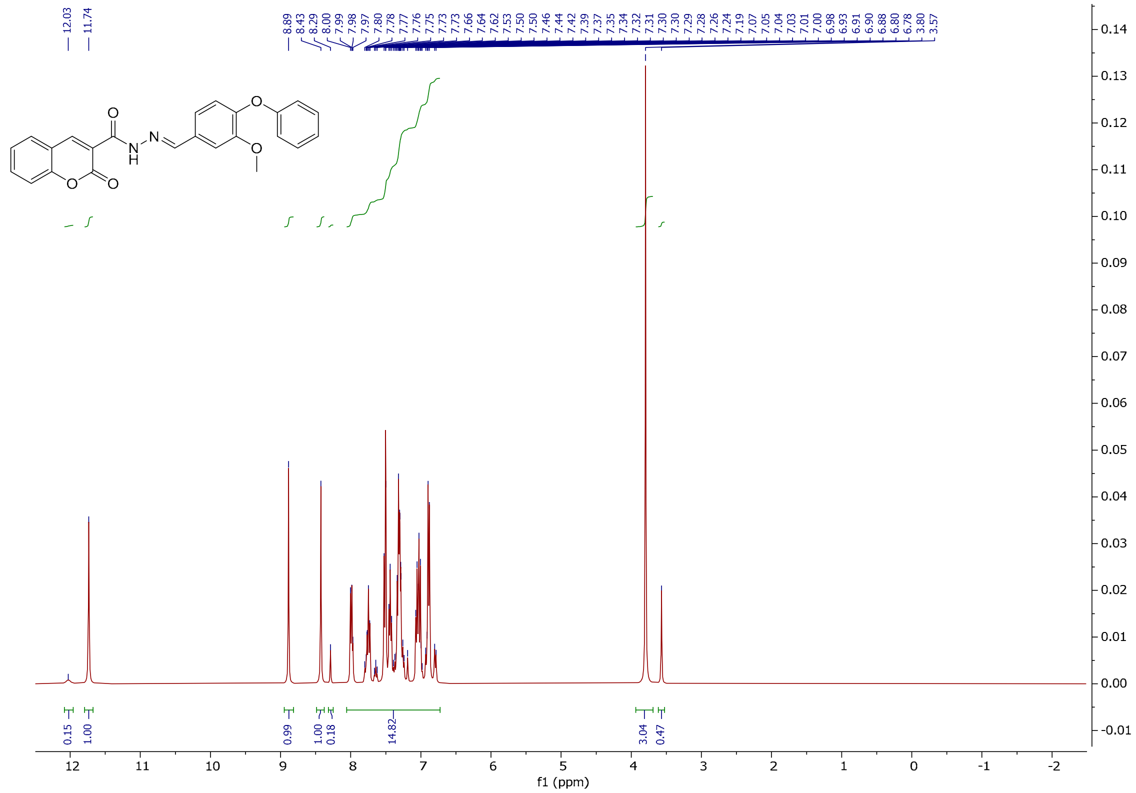

Figure S2. ^{13}C NMR spectra of *(E)-N′-(3-methoxy-4-phenoxybenzylidene)-2-oxo-2H-chromene-3-carbohydrazide* (**1**).

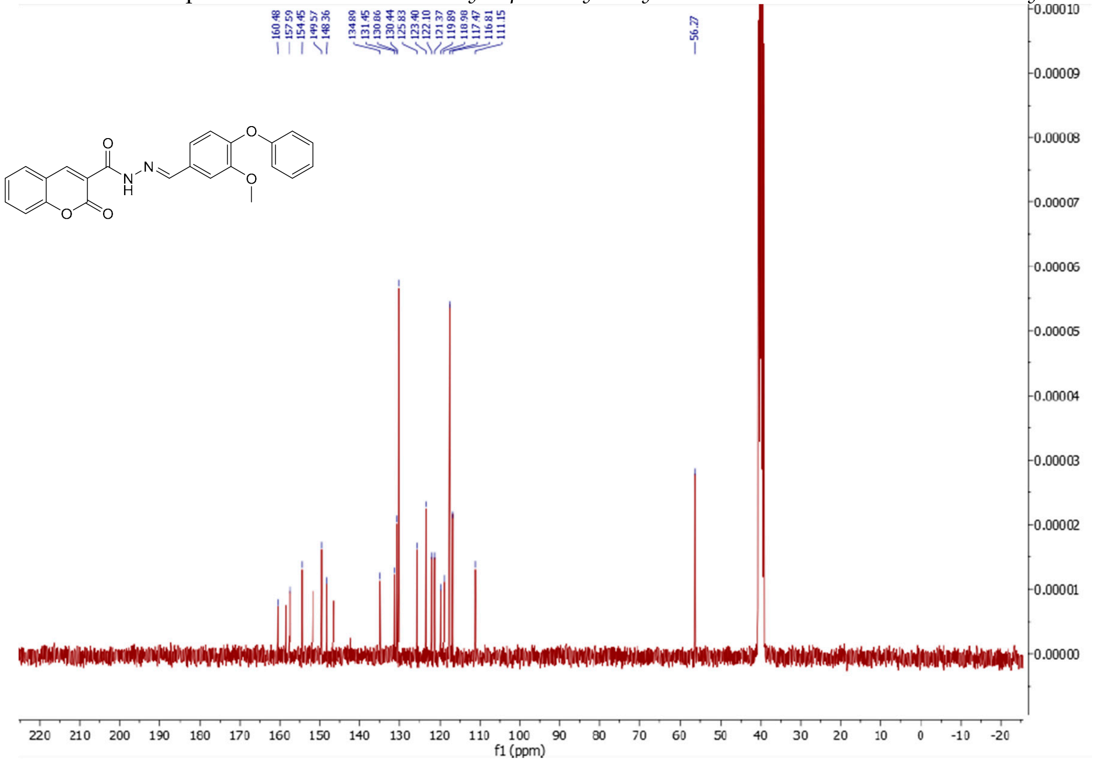

Figure S3. ^{1}H NMR spectra of *(E)-N′-(4-(benzyloxy)-3-methoxybenzylidene)-2-oxo-2H-chromene-3-carbohydrazide* (**2**).

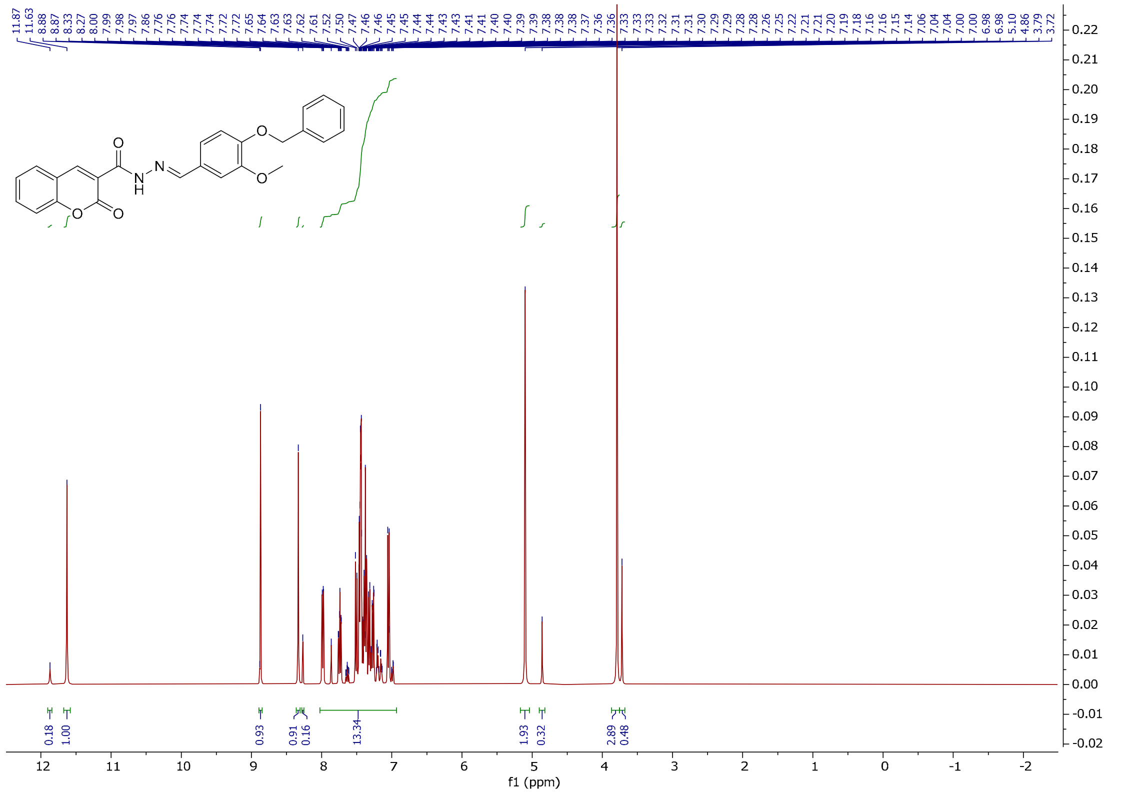

Figure S4. ^{13}C NMR spectra of *(E)-N′-(4-(benzyloxy)-3-methoxybenzylidene)-2-oxo-2H-chromene-3-carbohydrazide* (**2**).

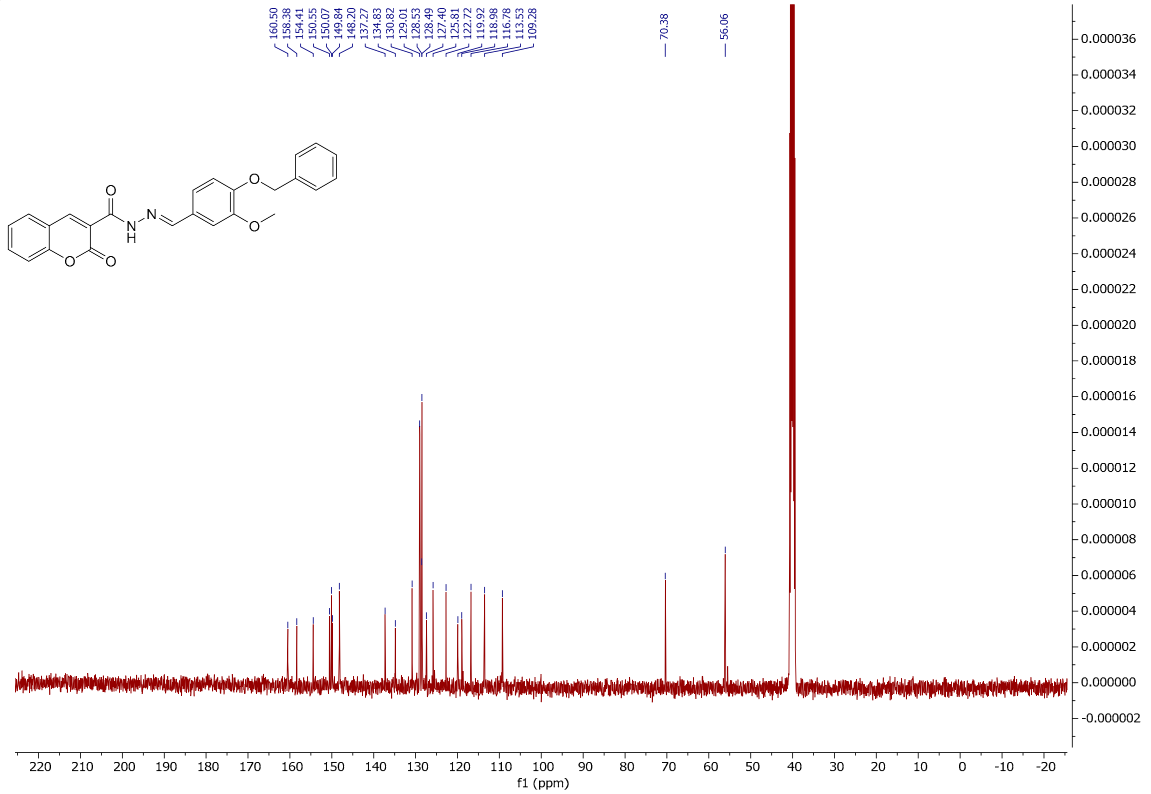

Figure S5. ^{1}H NMR spectra of *(E)-N′-(4-((4-fluorobenzyl)oxy)-3-methoxybenzylidene)-2-oxo-2H-chromene-3-carbohydrazide* (**3**).

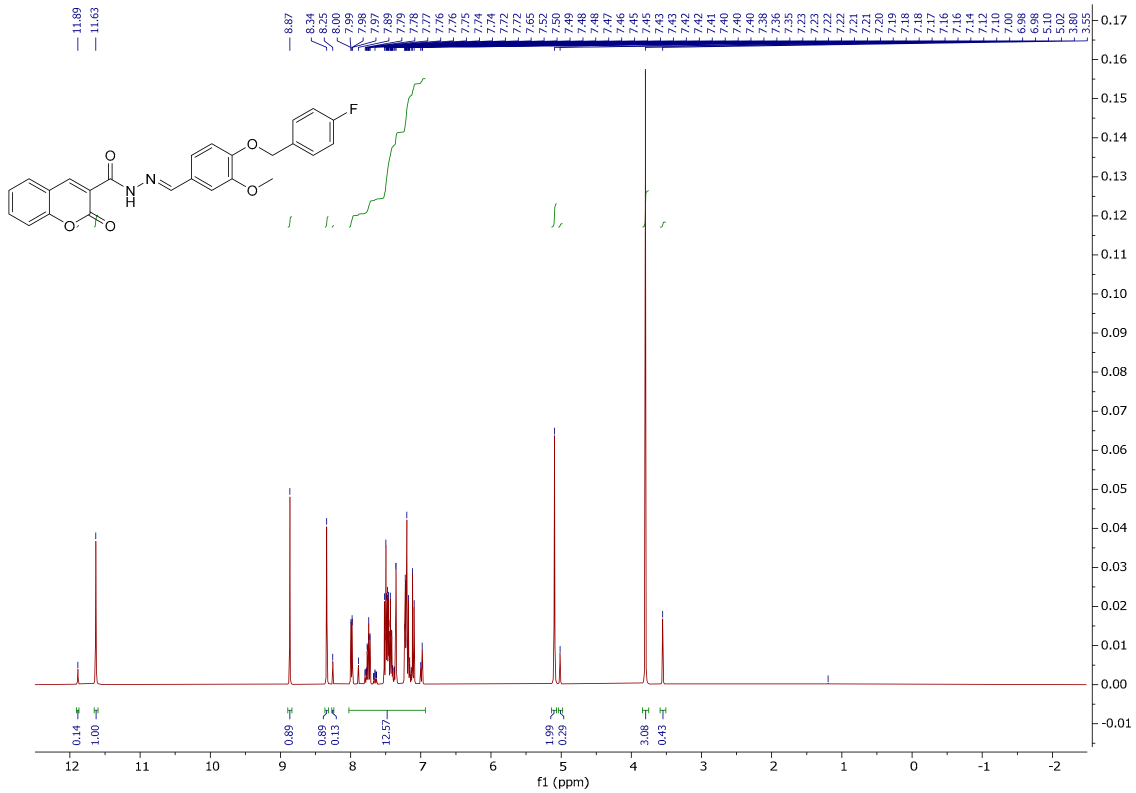

Figure S6. ^{13}C NMR spectra of *(E)-N′-(4-((4-fluorobenzyl)oxy)-3-methoxybenzylidene)-2-oxo-2H-chromene-3-carbohydrazide* (**3**).

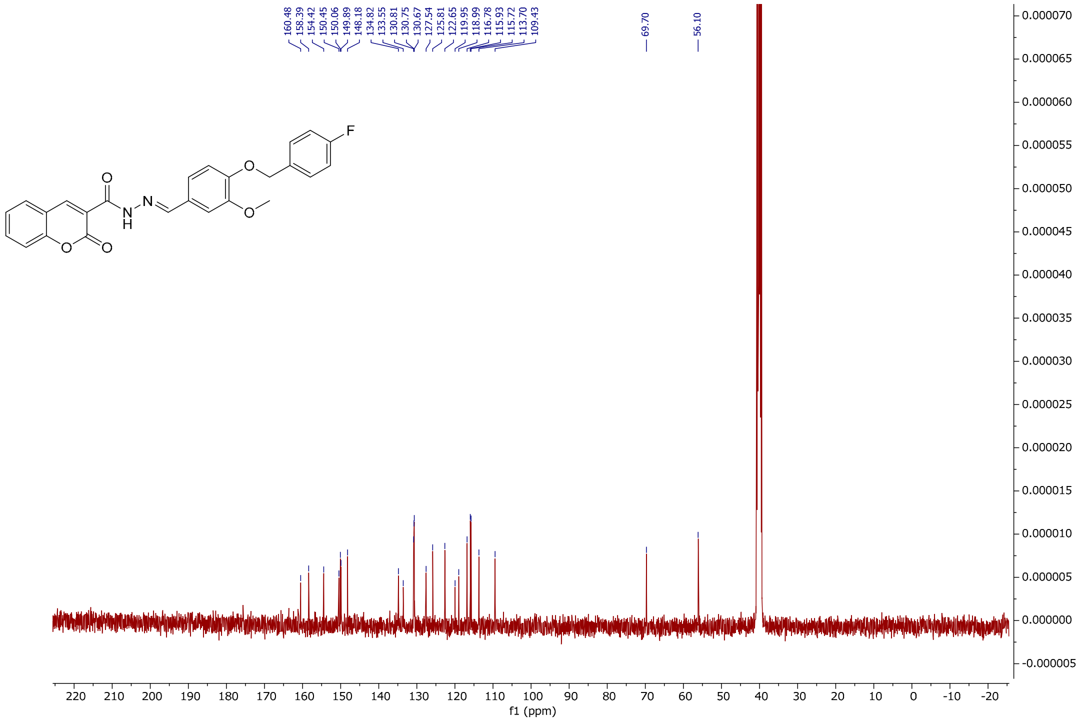

Figure S7. ^{1}H NMR spectra of *(E)-N′-(4-methoxy-3-phenoxybenzylidene)-2-oxo-2H-chromene-3-carbohydrazide* (**4**).

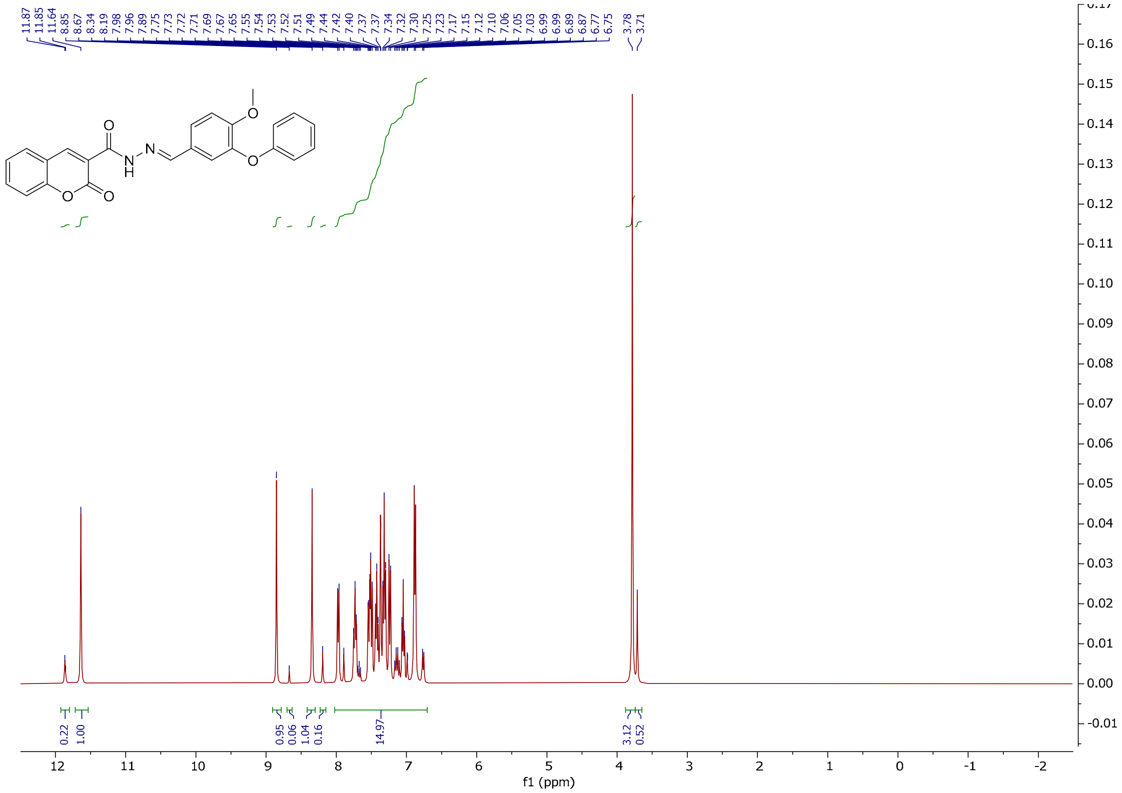

Figure S8. ^{13}C NMR spectra of *(E)-N′-(4-methoxy-3-phenoxybenzylidene)-2-oxo-2H-chromene-3-carbohydrazide* (**4**).

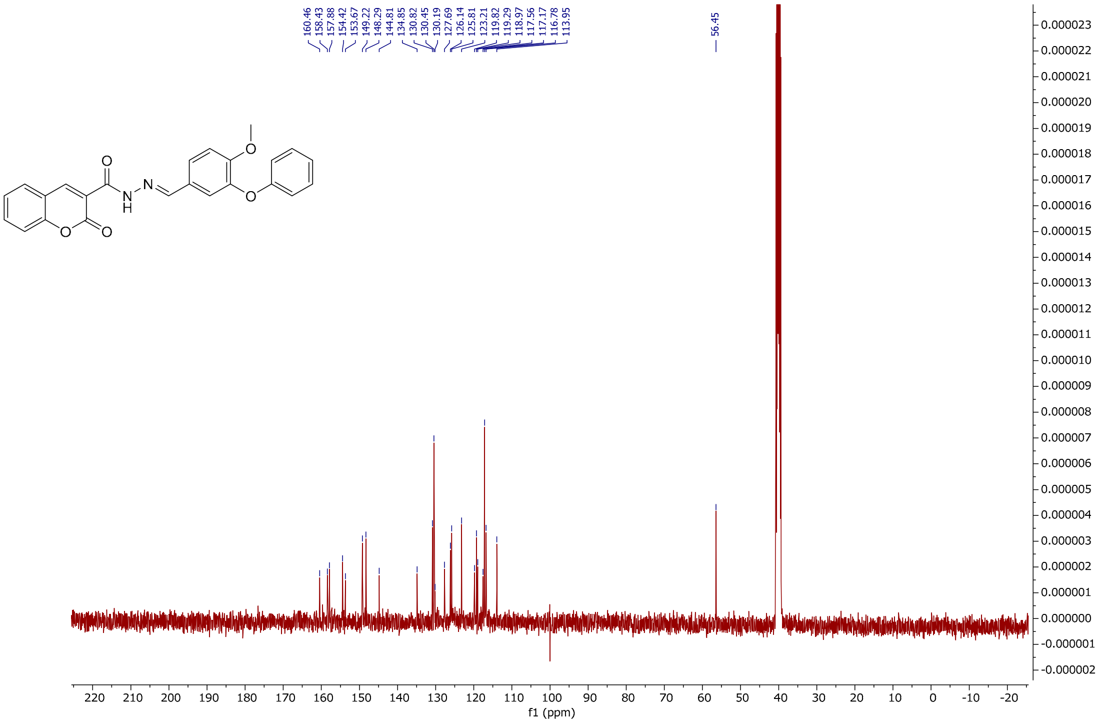

Figure S9. ^{1}H NMR spectra of *(E)-N′-(3-(benzyloxy)-4-methoxybenzylidene)-2-oxo-2H-chromene-3-carbohydrazide* (**5**).

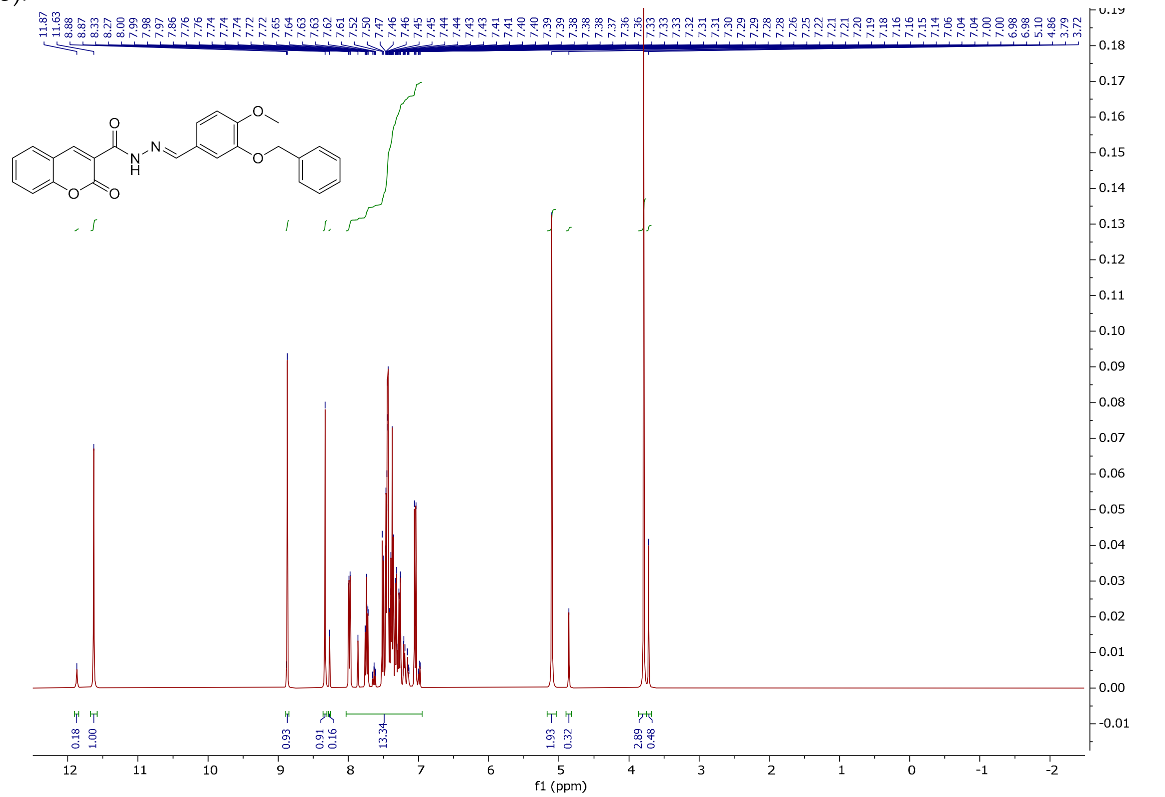

Figure S10. ^{13}C NMR spectra of *(E)-N′-(3-(benzyloxy)-4-methoxybenzylidene)-2-oxo-2H-chromene-3-carbohydrazide* (**5**).

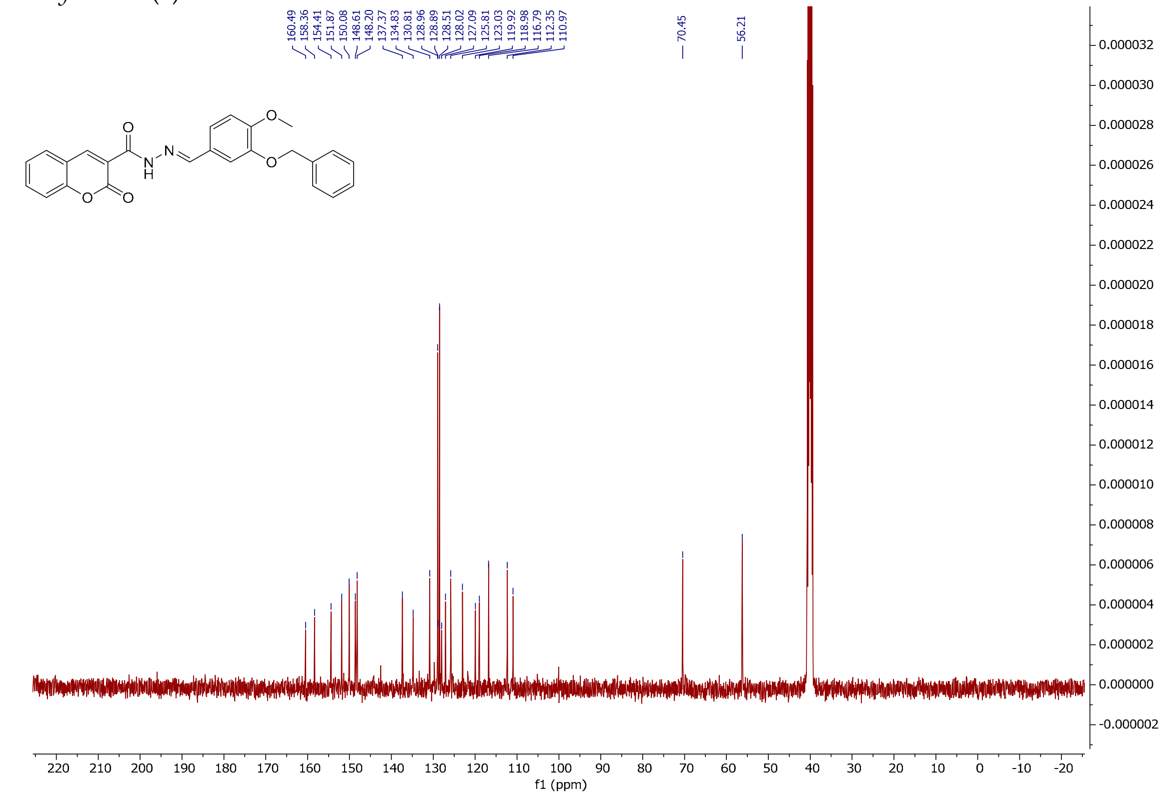

Figure S11. ^{1}H NMR spectra of *(E)-N′-(3-((4-fluorobenzyl)oxy)-4-methoxybenzylidene)-2-oxo-2H-chromene-3-carbohydrazide* (**6**).

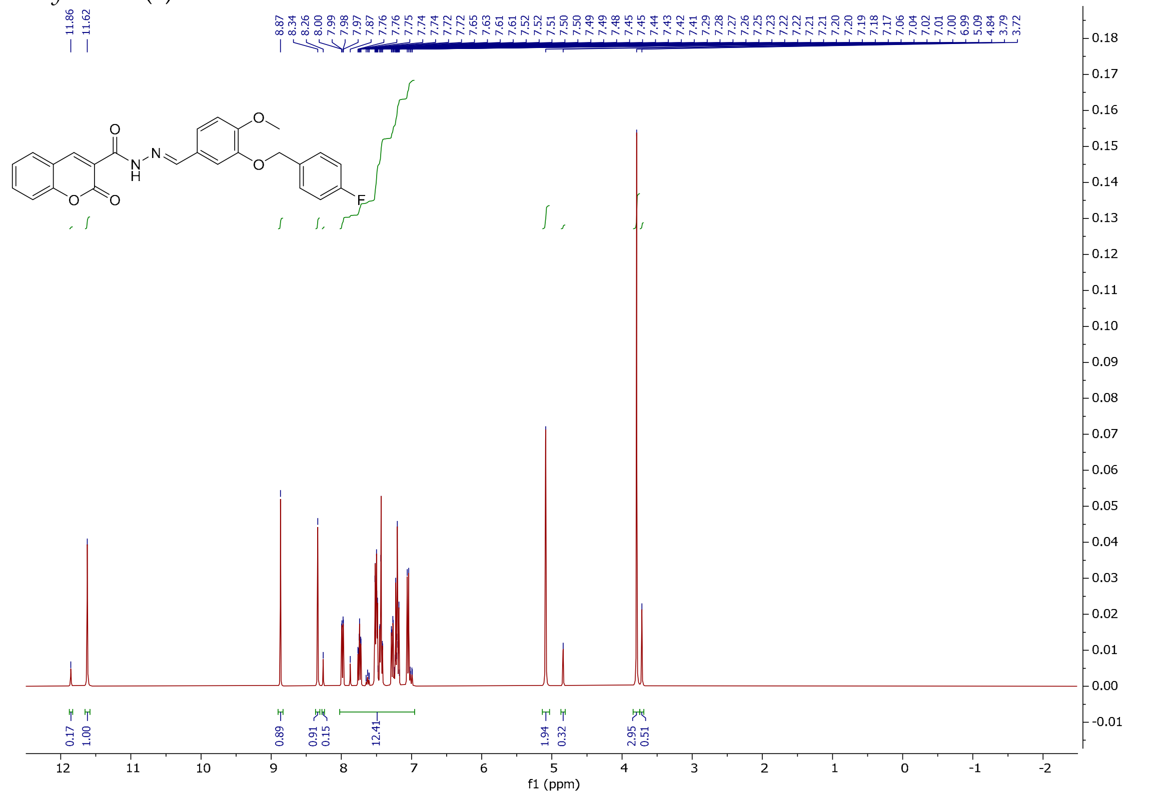

Figure S12. ^{13}C NMR spectra of *(E)-N′-(3-((4-fluorobenzyl)oxy)-4-methoxybenzylidene)-2-oxo-2H-chromene-3-carbohydrazide* (**6**).

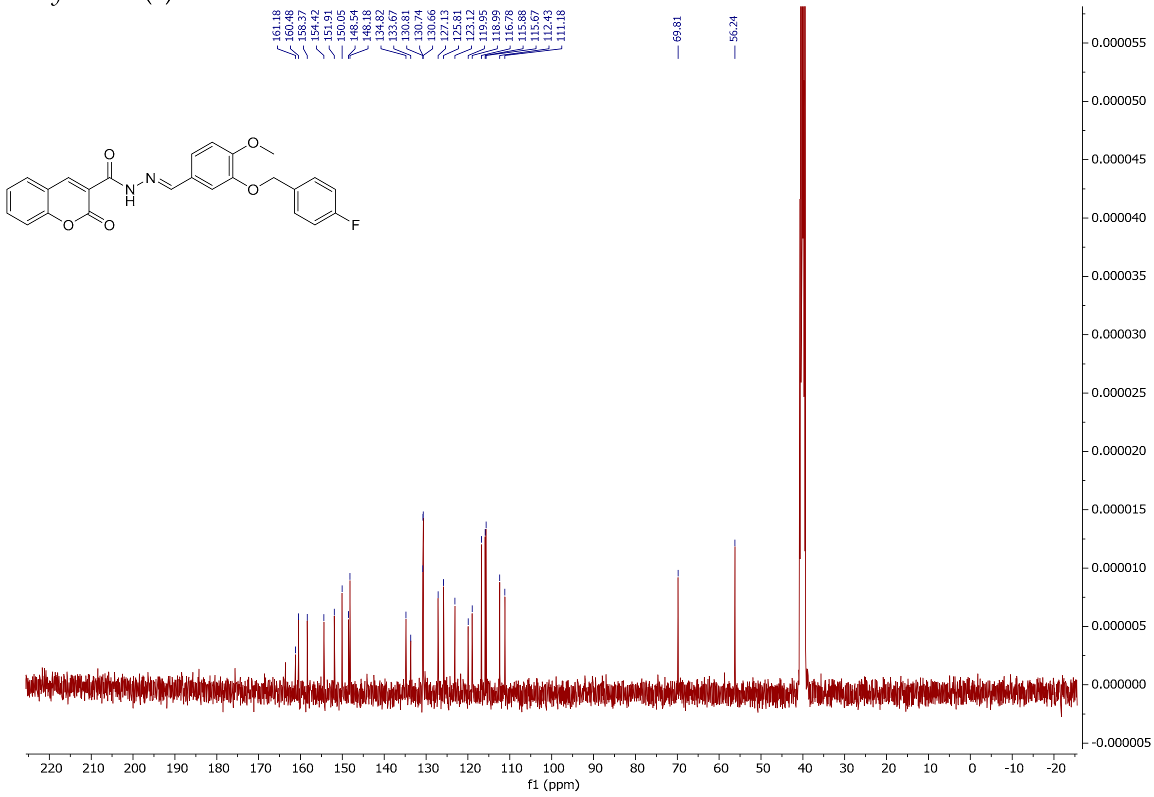

Figure S13. ^{1}H NMR spectra of *(E)-8-methoxy-N′-(3-methoxy-4-phenoxybenzylidene)-2-oxo-2H-chromene-3-carbohydrazide* (**7**).

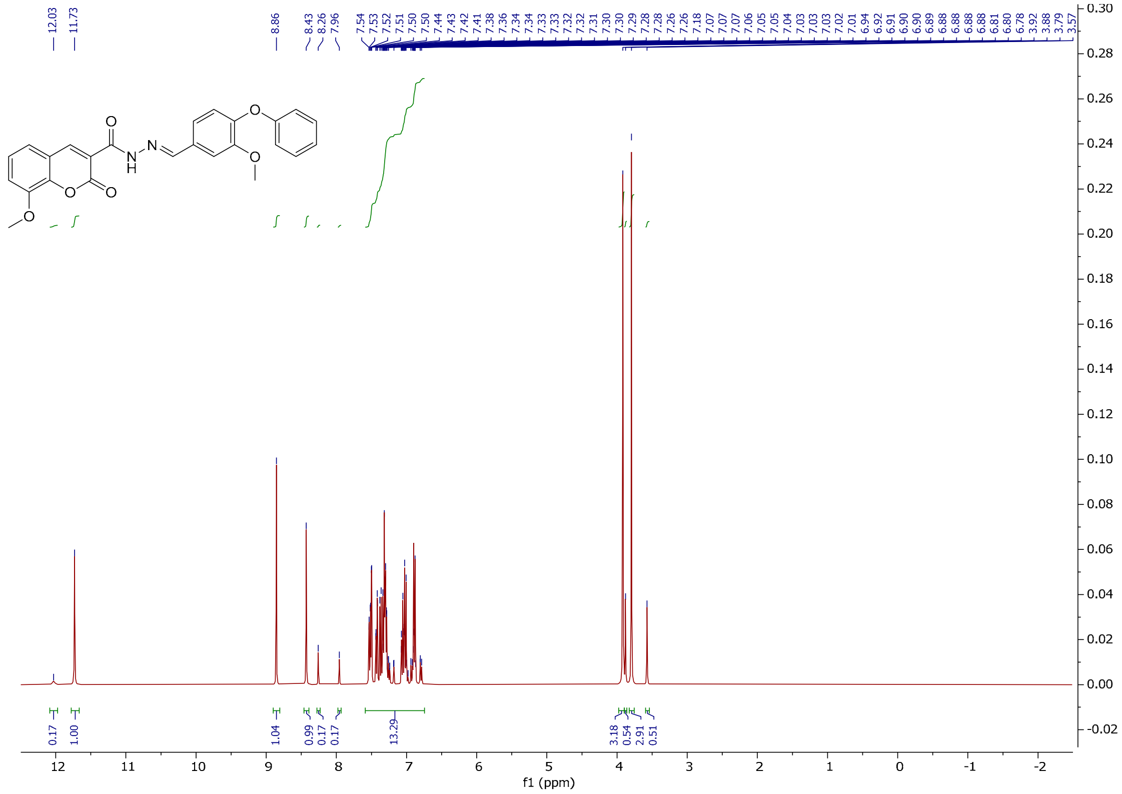

Figure S14. ^{13}C NMR spectra of *(E)-8-methoxy-N′-(3-methoxy-4-phenoxybenzylidene)-2-oxo-2H-chromene-3-carbohydrazide* (**7**).

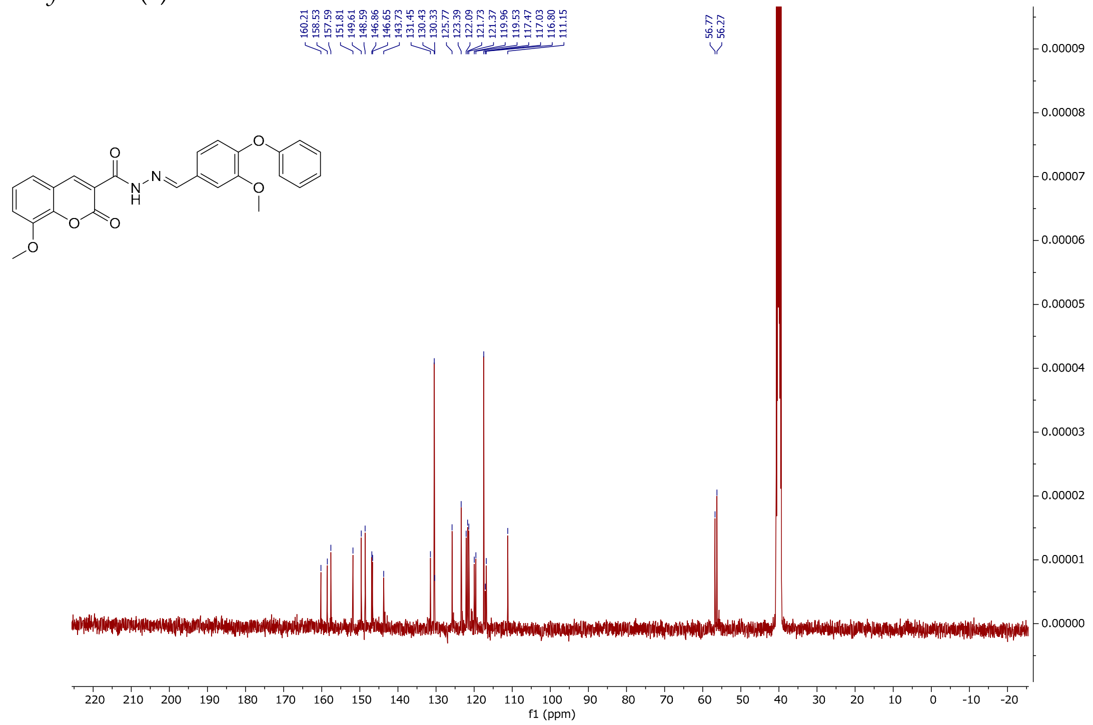

Figure S15. ^{1}H NMR spectra of *(E)-N′-(4-(benzyloxy)-3-methoxybenzylidene)-8-methoxy-2-oxo-2H-chromene-3-carbohydrazide* (**8**).

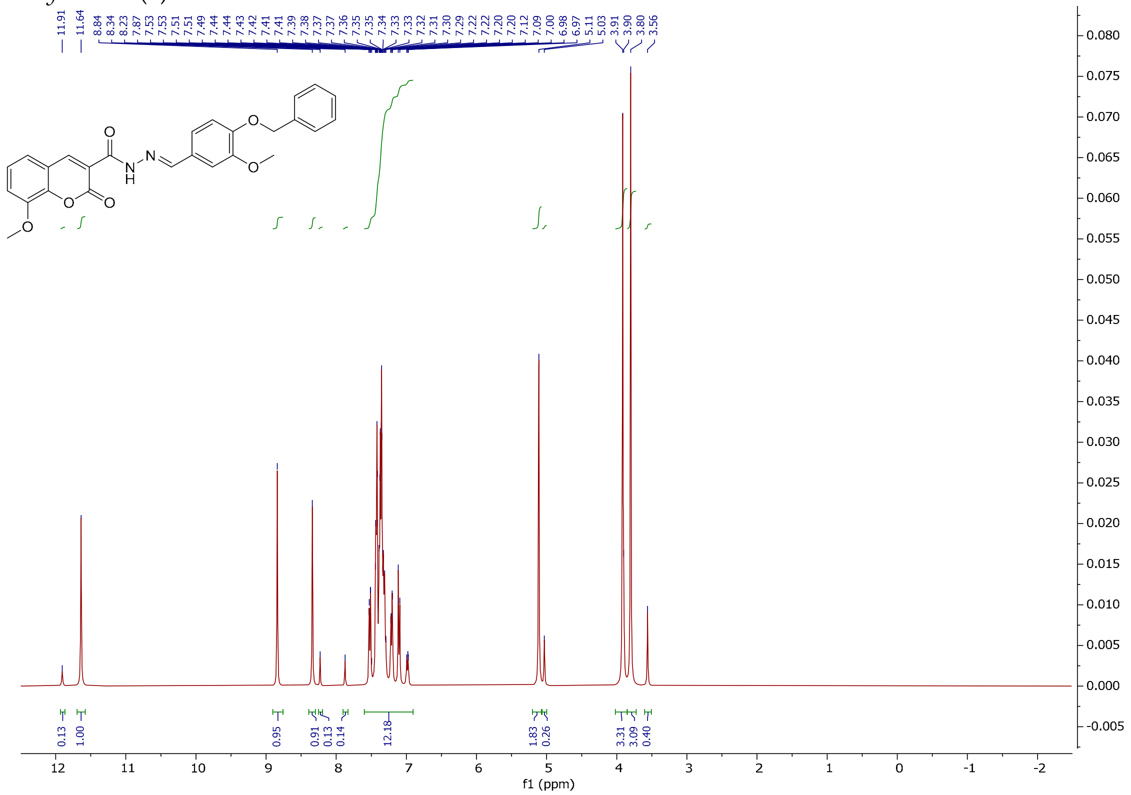

Figure S16. ^{13}C NMR spectra of *(E)-N′-(4-(benzyloxy)-3-methoxybenzylidene)-8-methoxy-2-oxo-2H-chromene-3-carbohydrazide* (**8**).

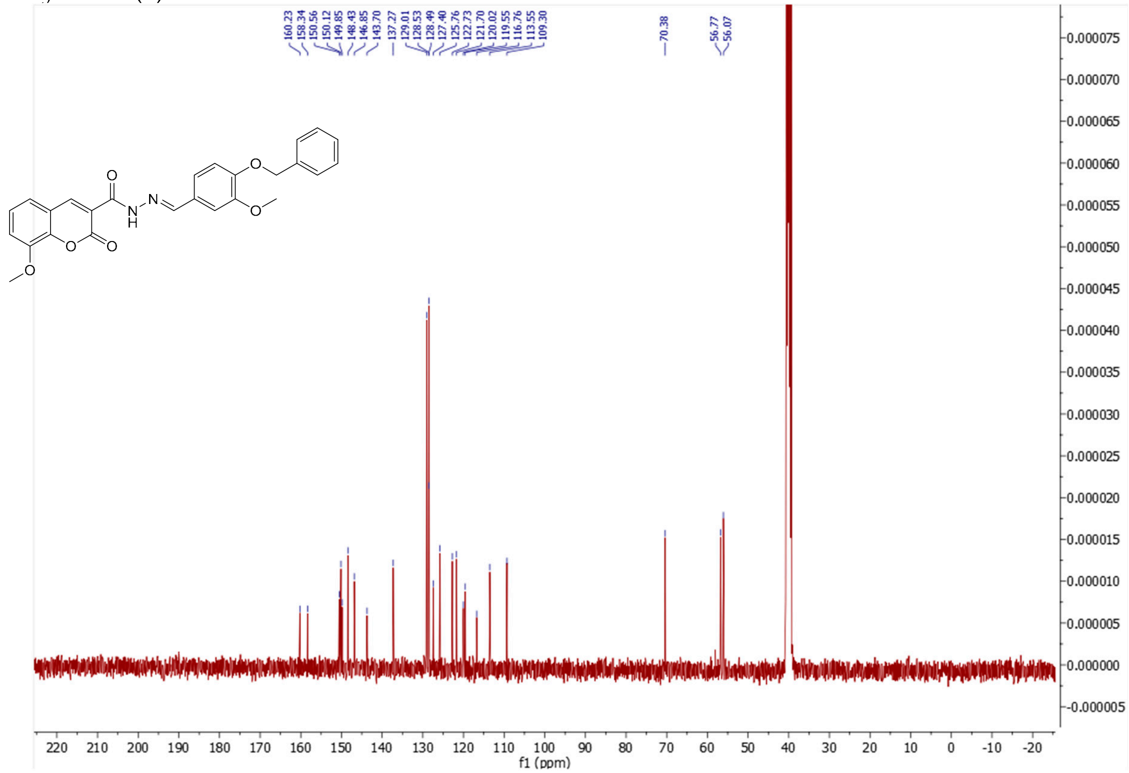

Figure S17. ^{1}H NMR spectra of *(E)-N′-(4-((4-fluorobenzyl)oxy)-3-methoxybenzylidene)-8-methoxy-2-oxo-2H-chromene-3-carbohydrazide* (**9**).

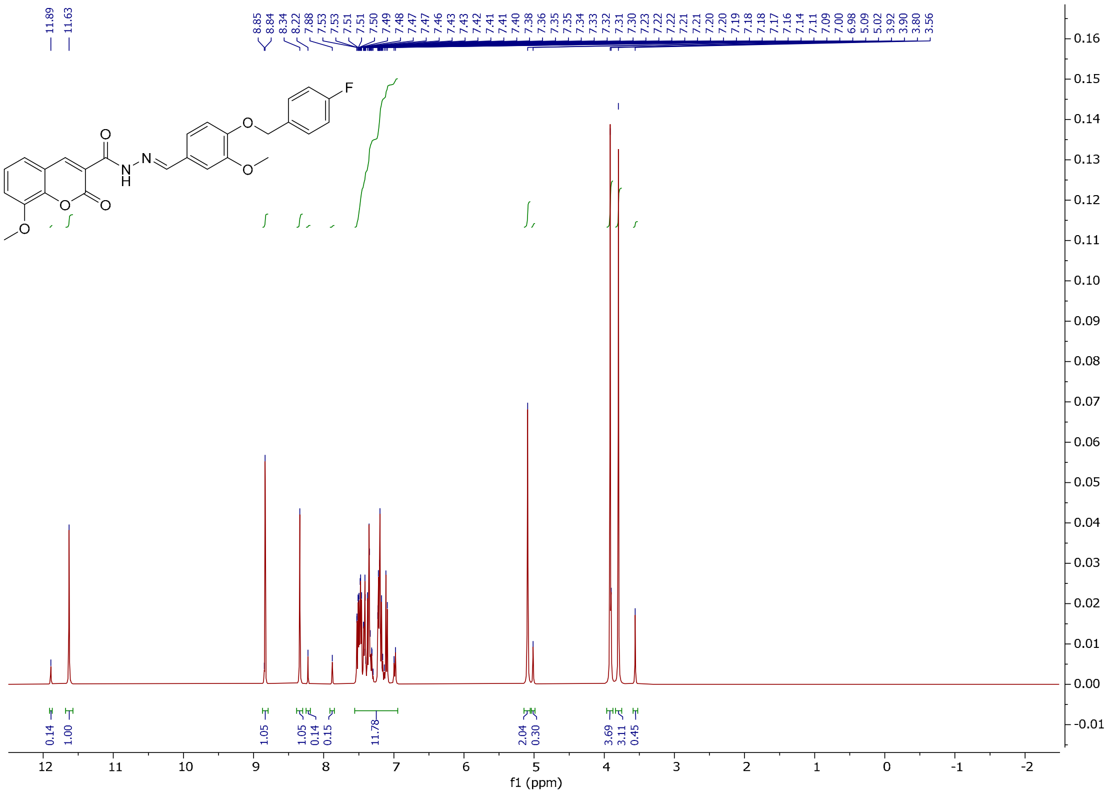

Figure S18. ^{13}C NMR spectra of *(E)-N′-(4-((4-fluorobenzyl)oxy)-3-methoxybenzylidene)-8-methoxy-2-oxo-2H-chromene-3-carbohydrazide* (**9**).

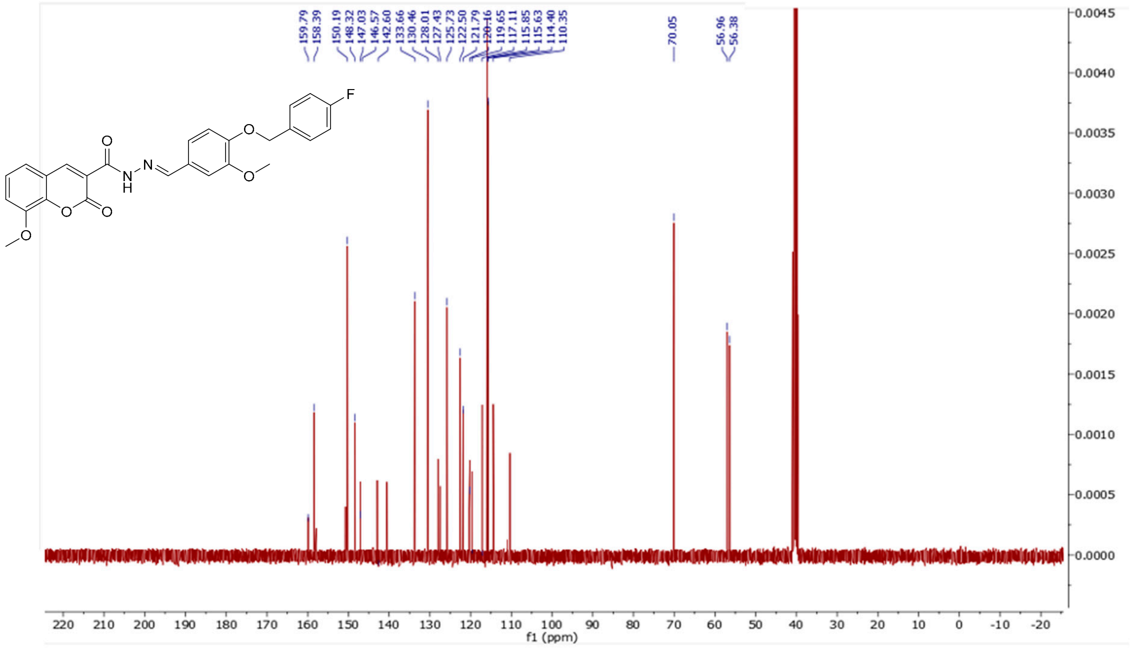

Figure S19. ^{1}H NMR spectra of *(E)-8-methoxy-N′-(4-methoxy-3-phenoxybenzylidene)-2-oxo-2H-chromene-3-carbohydrazide* (**10**).

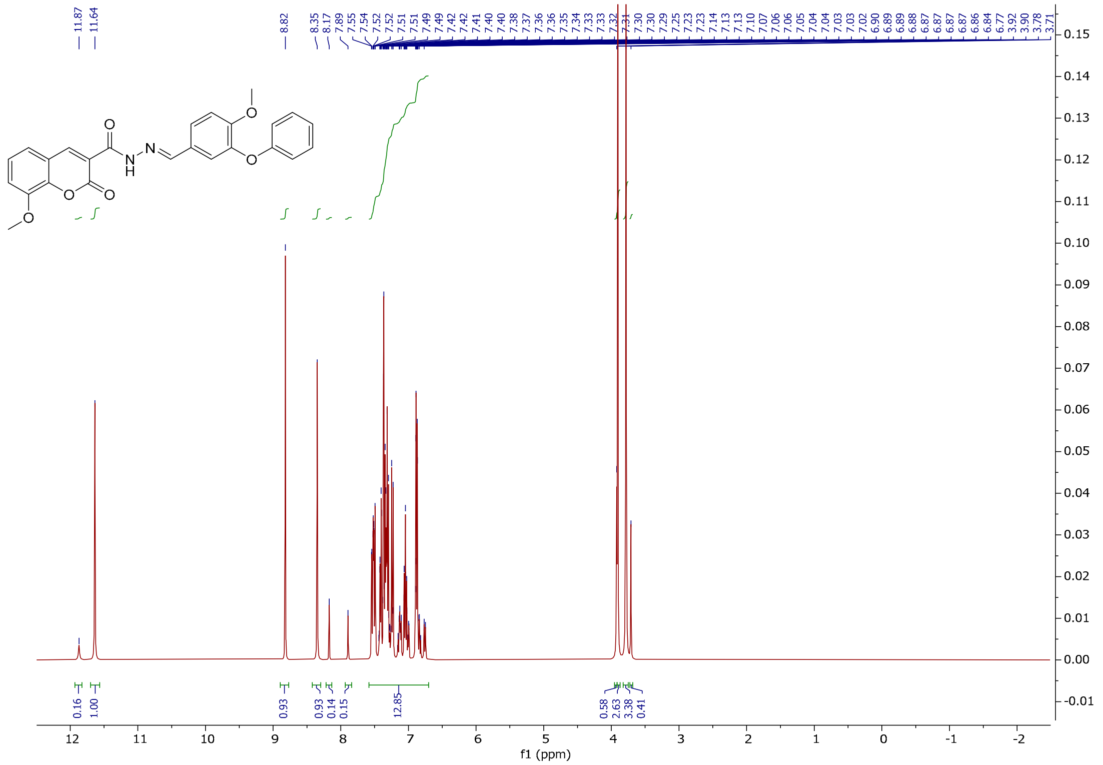

Figure S20. ^{13}C NMR spectra of *(E)-8-methoxy-N′-(4-methoxy-3-phenoxybenzylidene)-2-oxo-2H-chromene-3-carbohydrazide* (**10**).

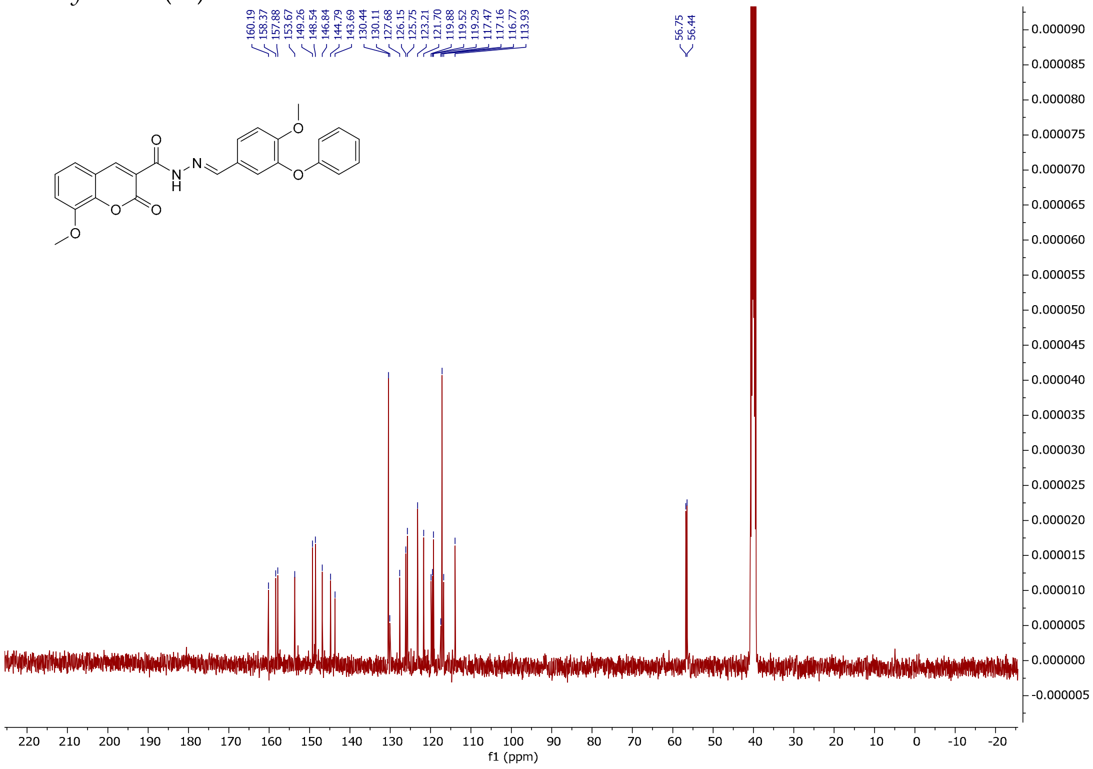

Figure S21. ^{1}H NMR spectra of *(E)-N′-(3-(benzyloxy)-4-methoxybenzylidene)-8-methoxy-2-oxo-2H-chromene-3-carbohydrazide* (**11**).

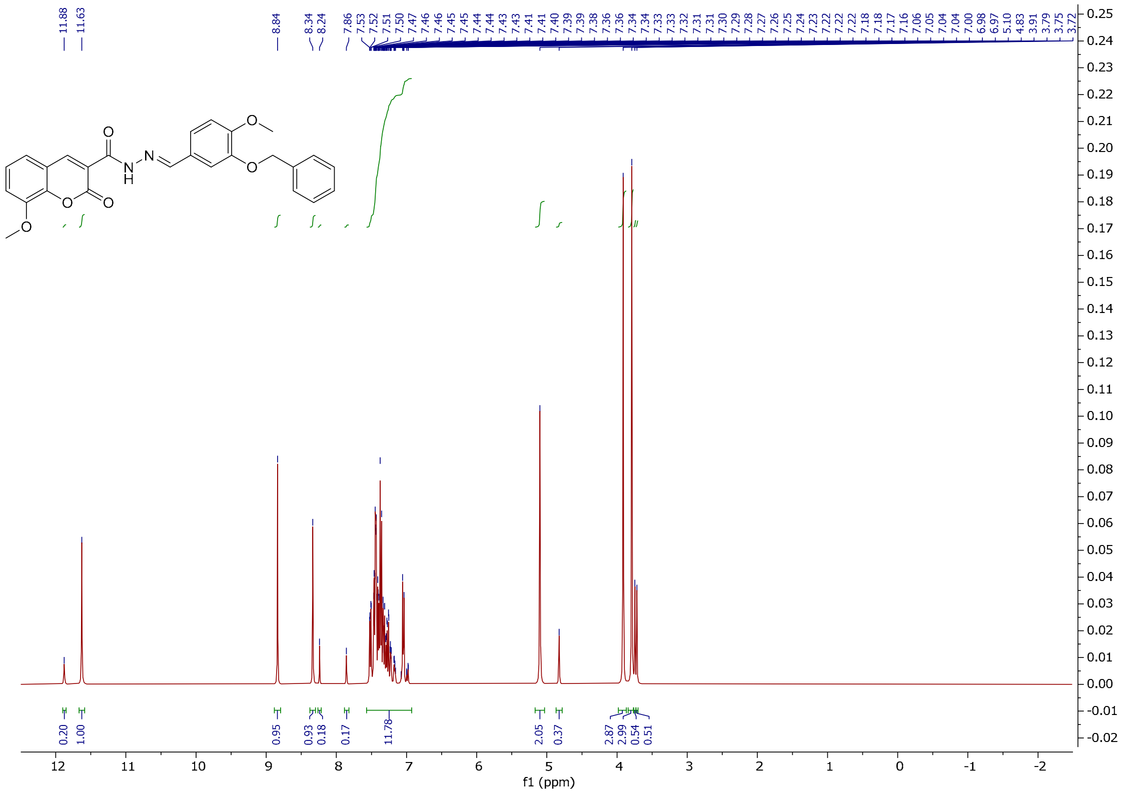

Figure S22. ^{13}C NMR spectra of *(E)-N′-(3-(benzyloxy)-4-methoxybenzylidene)-8-methoxy-2-oxo-2H-chromene-3-carbohydrazide* (**11**).

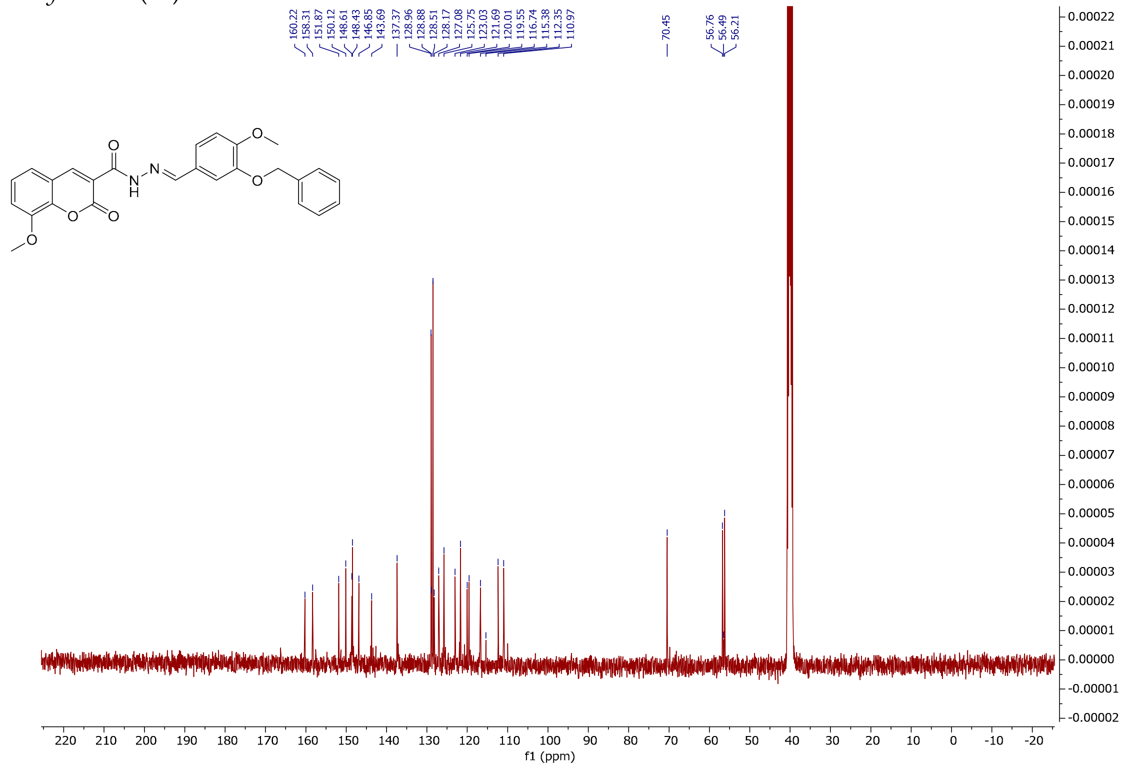

Figure S23. ^{1}H NMR spectra of *(E)-N′-(3-((4-fluorobenzyl)oxy)-4-methoxybenzylidene)-8-methoxy-2-oxo-2H-chromene-3-carbohydrazide* (**12**).

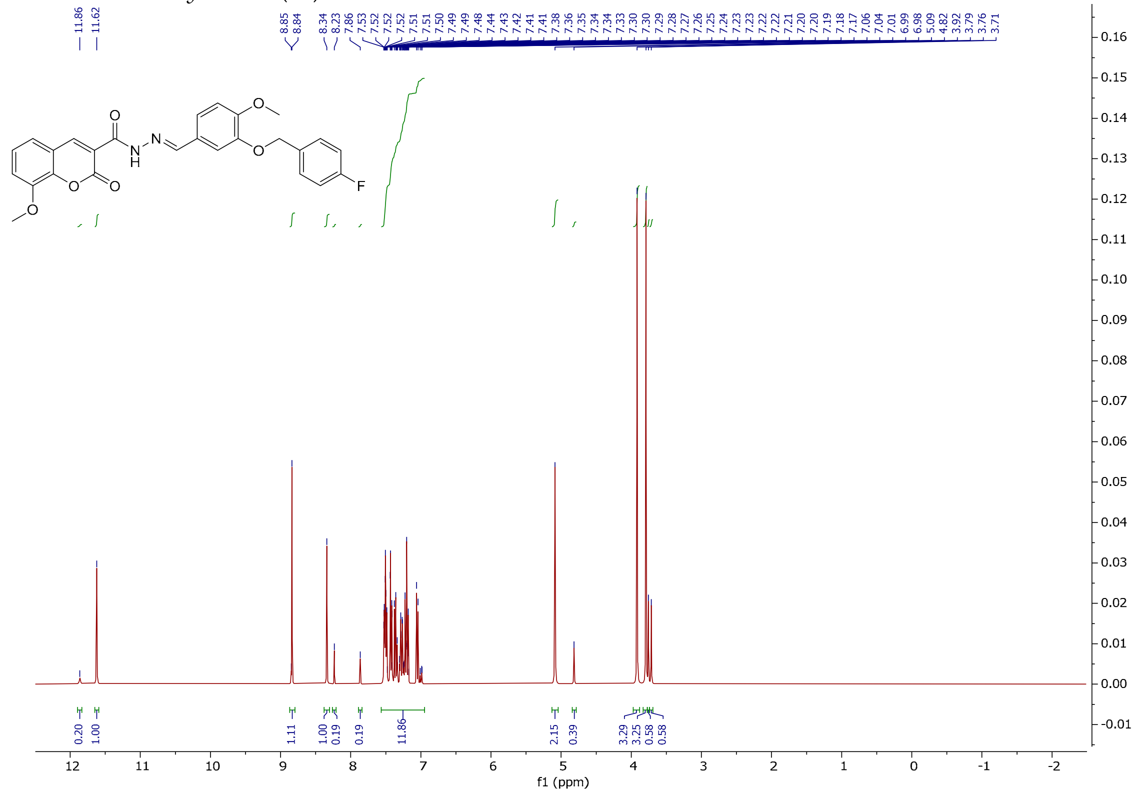

Figure S24. ^{13}C NMR spectra of *(E)-N′-(3-((4-fluorobenzyl)oxy)-4-methoxybenzylidene)-8-methoxy-2-oxo-2H-chromene-3-carbohydrazide* (**12**).

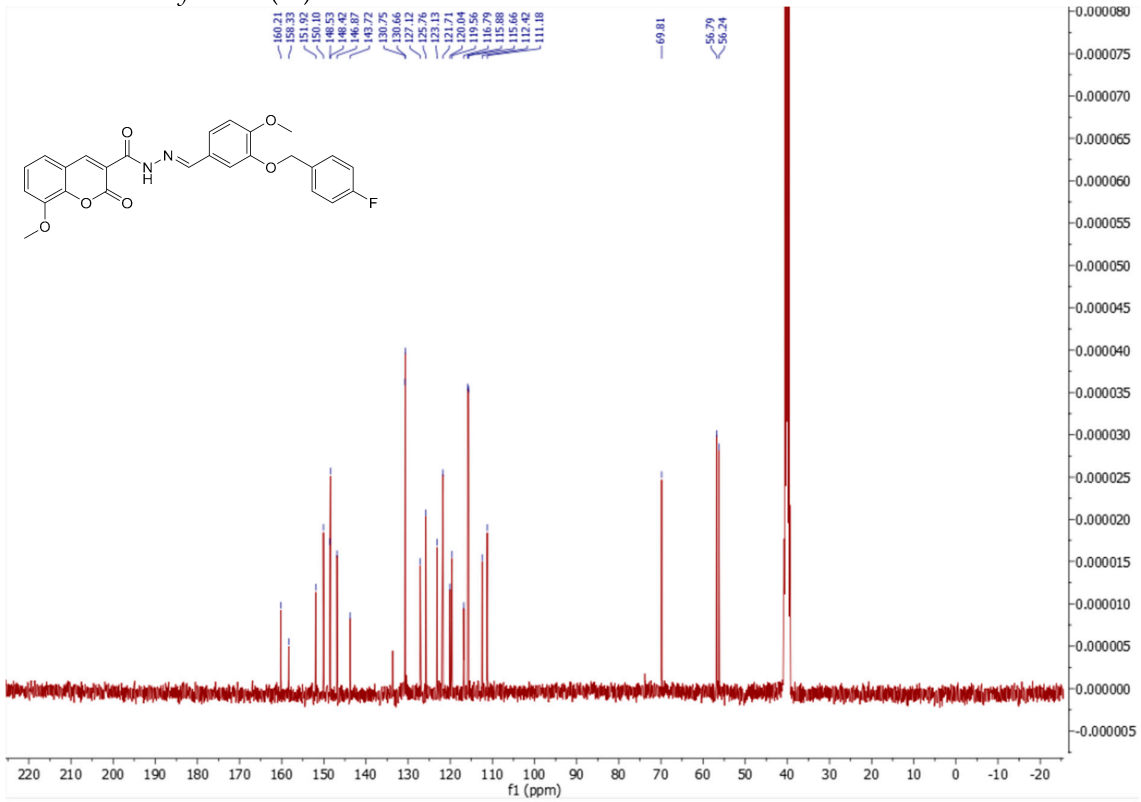

Figure S25. ^{1}H NMR spectra of *(E)-8-hydroxy-N′-(3-methoxy-4-phenoxybenzylidene)-2-oxo-2H-chromene-3-carbohydrazide* (**13**).

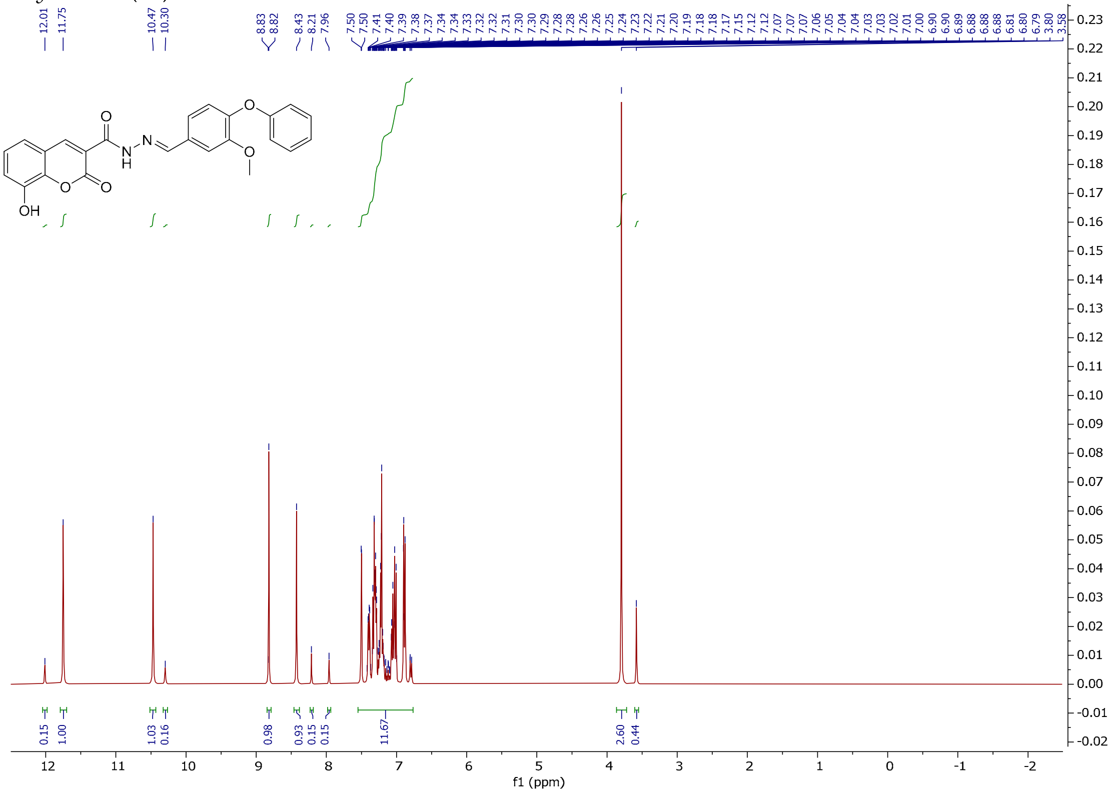

Figure S26. ^{13}C NMR spectra of *(E)-8-hydroxy-N′-(3-methoxy-4-phenoxybenzylidene)-2-oxo-2H-chromene-3-carbohydrazide* (**13**).

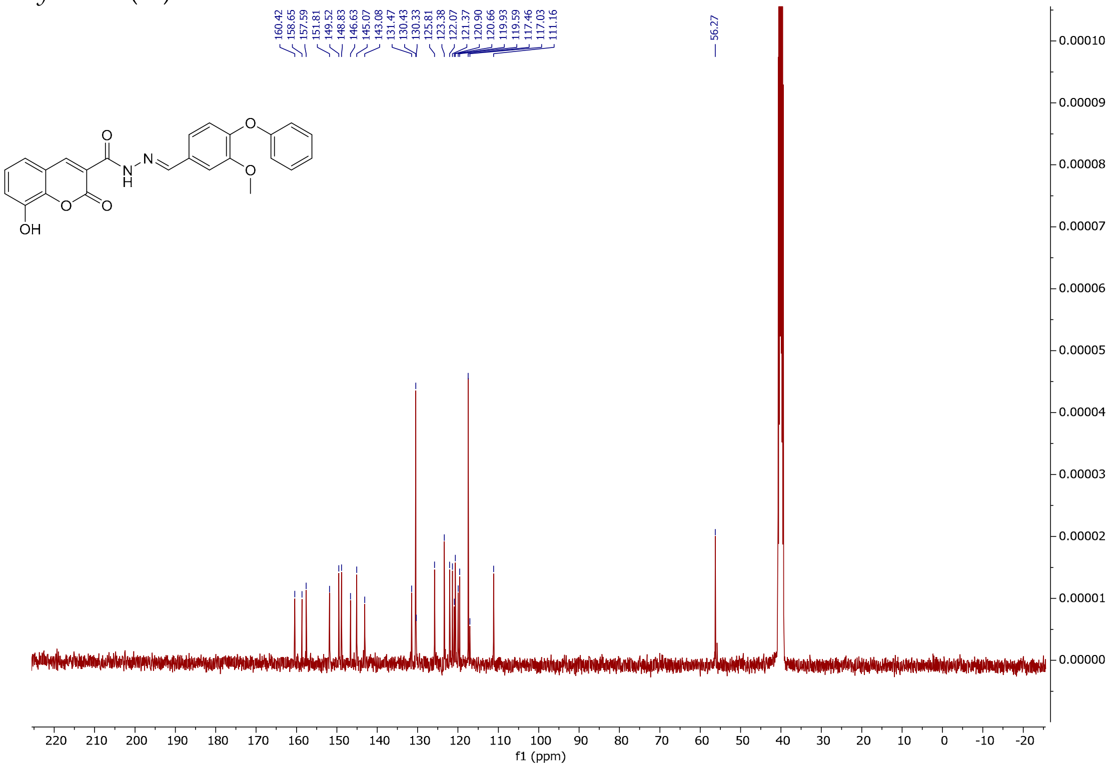

Figure S27. ^{1}H NMR spectra of *(E)-8-hydroxy-N′-(4-methoxy-3-phenoxybenzylidene)-2-oxo-2H-chromene-3-carbohydrazide* (**14**).

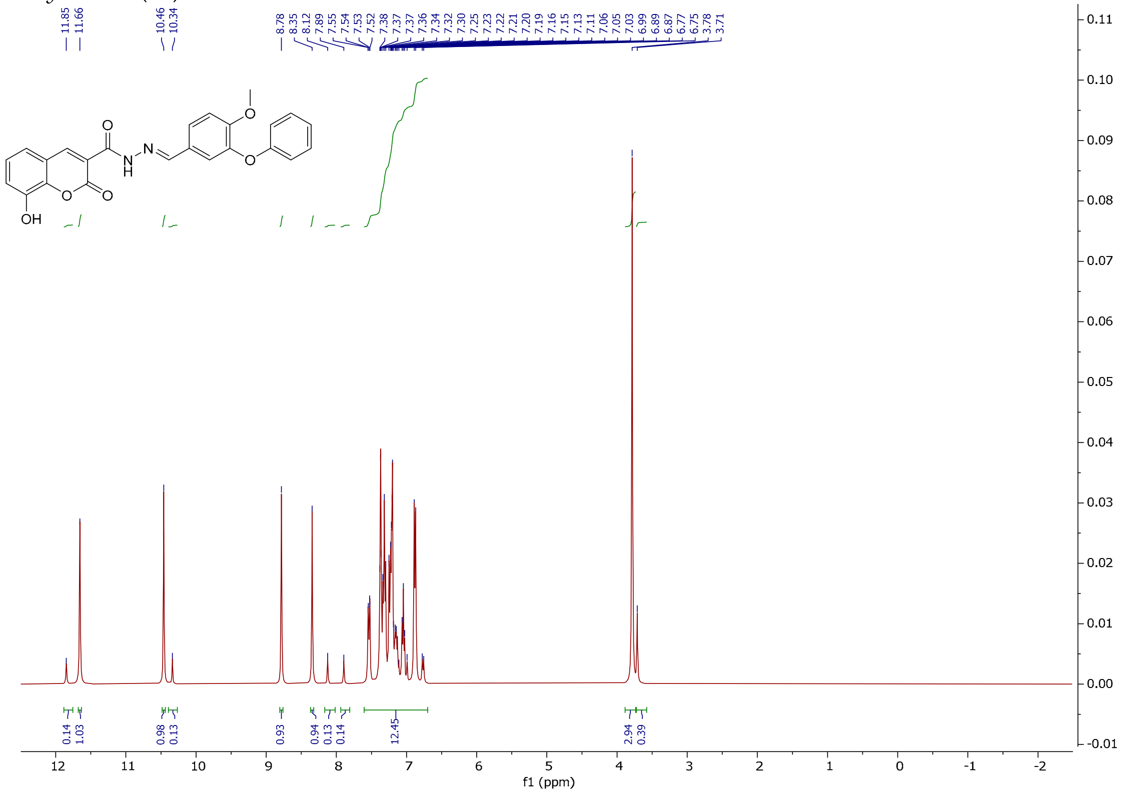

Figure S28. ^{13}C NMR spectra of *(E)-8-hydroxy-N′-(4-methoxy-3-phenoxybenzylidene)-2-oxo-2H-chromene-3-carbohydrazide* (**14**).

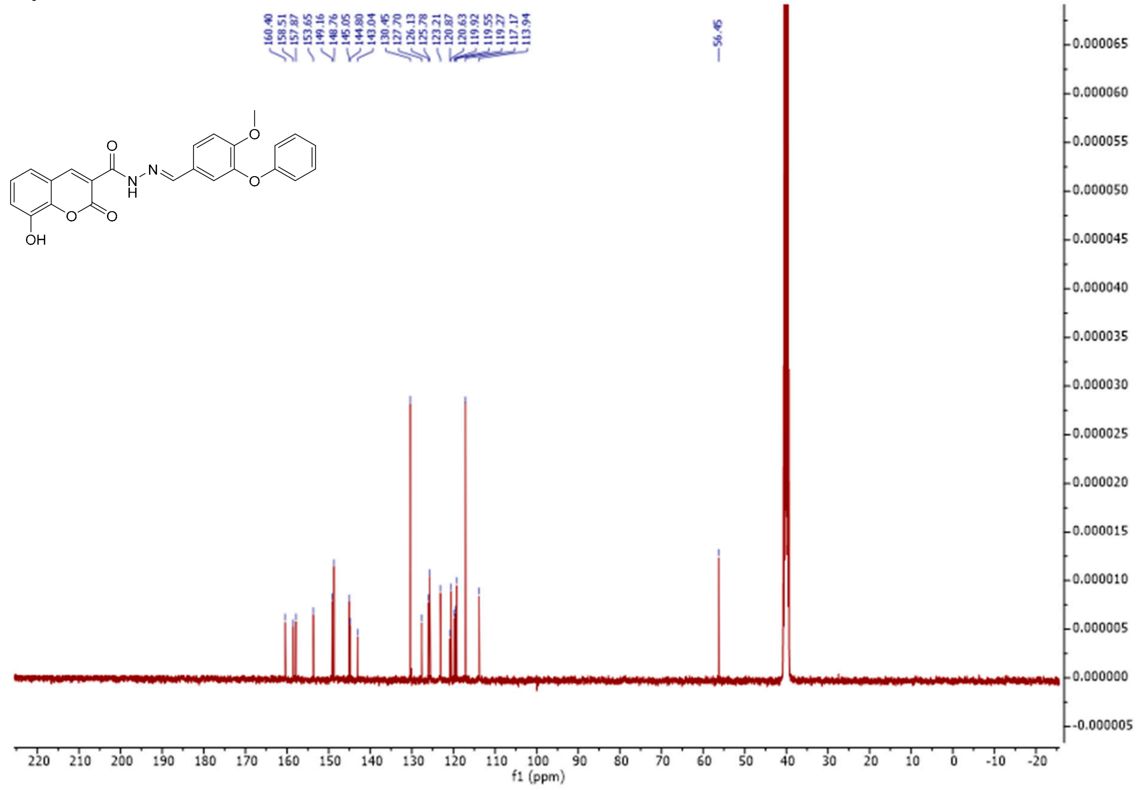

Figure S29. ^{1}H NMR spectra of *(E)-N′-(3-(benzyloxy)-4-methoxybenzylidene)-8-hydroxy-2-oxo-2H-chromene-3-carbohydrazide* (**15**).

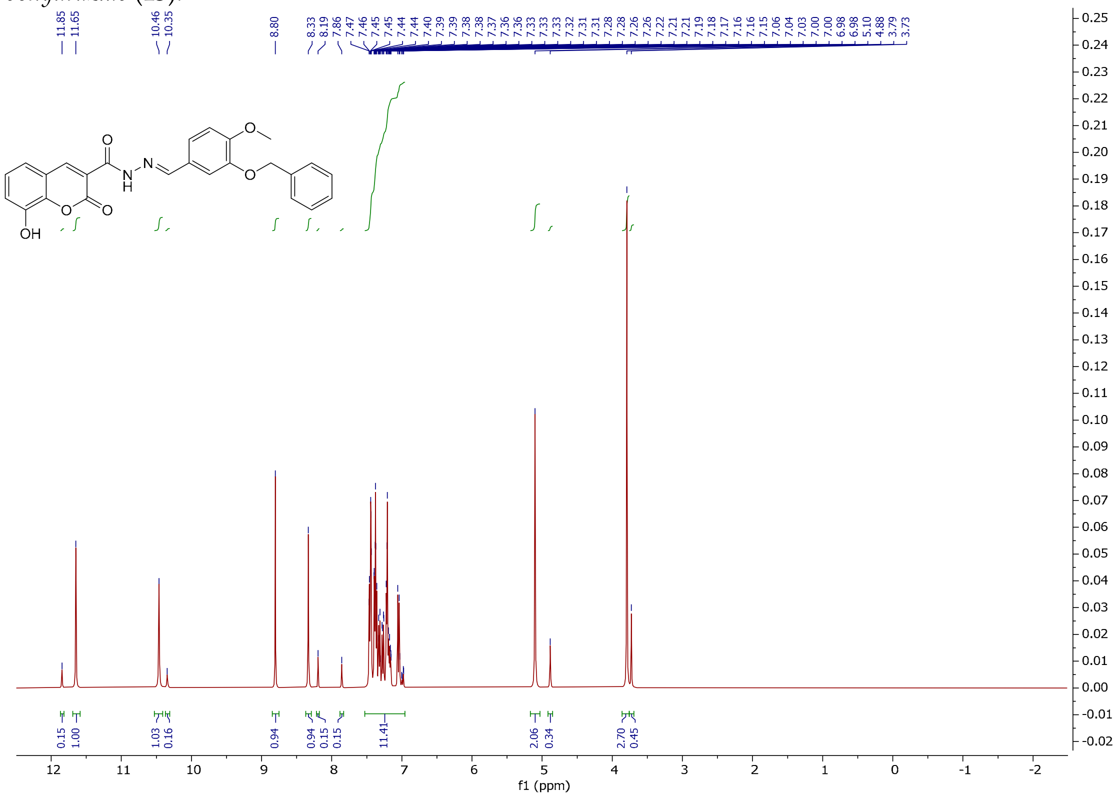

Figure S30. ^{13}C NMR spectra of *(E)-N′-(3-(benzyloxy)-4-methoxybenzylidene)-8-hydroxy-2-oxo-2H-chromene-3-carbohydrazide* (**15**).

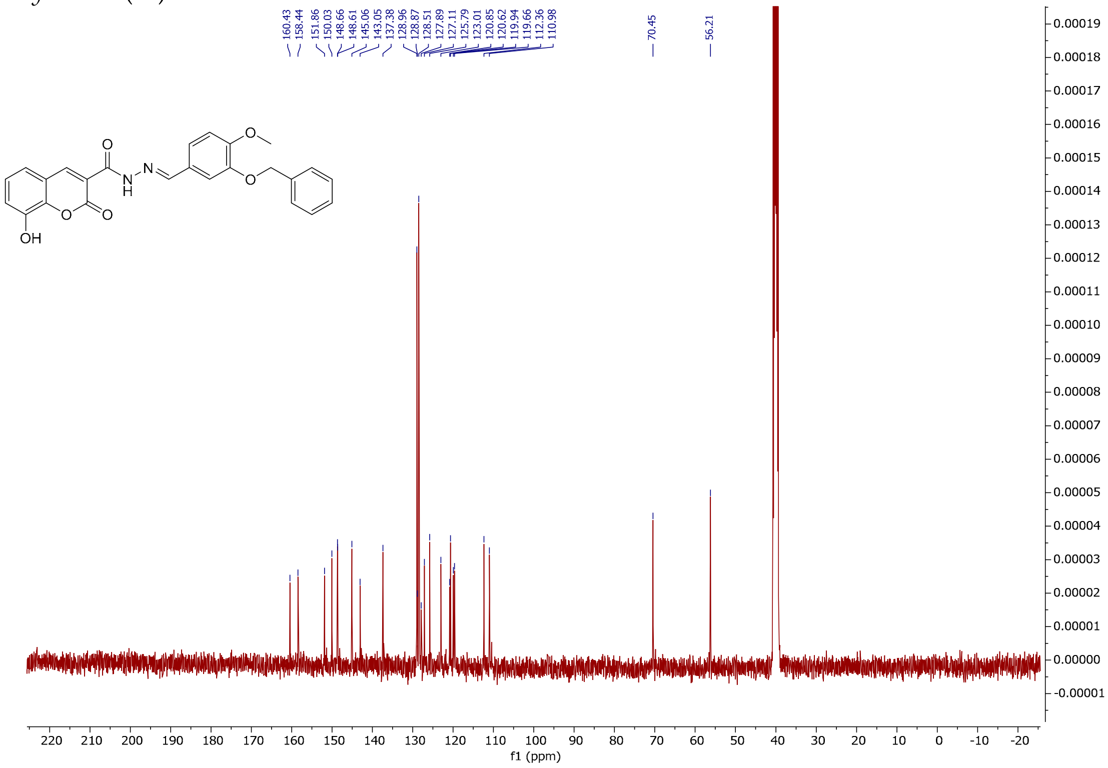

Figure S31. ^{1}H NMR spectra of *(E)-N′-(3-((4-fluorobenzyl)oxy)-4-methoxybenzylidene)-8-hydroxy-2-oxo-2H-chromene-3-carbohydrazide* (**16**).

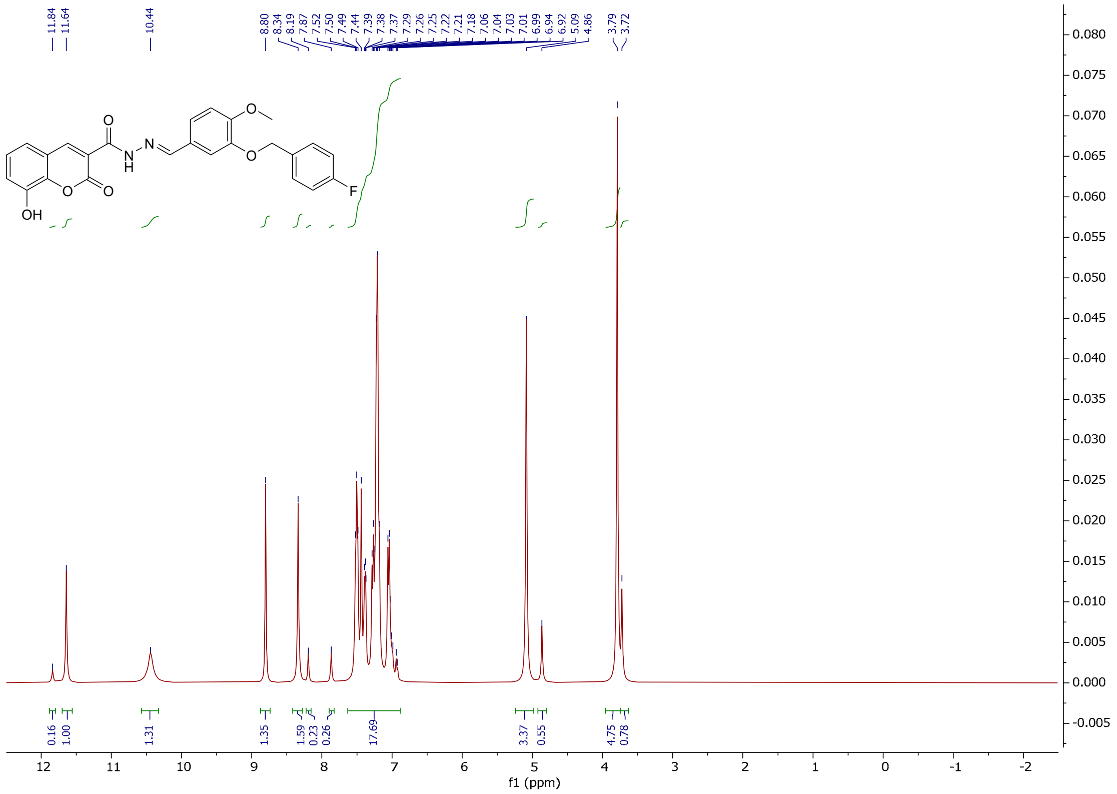

Figure S32. ^{13}C NMR spectra of *(E)-N′-(3-((4-fluorobenzyl)oxy)-4-methoxybenzylidene)-8-hydroxy-2-oxo-2H-chromene-3-carbohydrazide* (**16**).

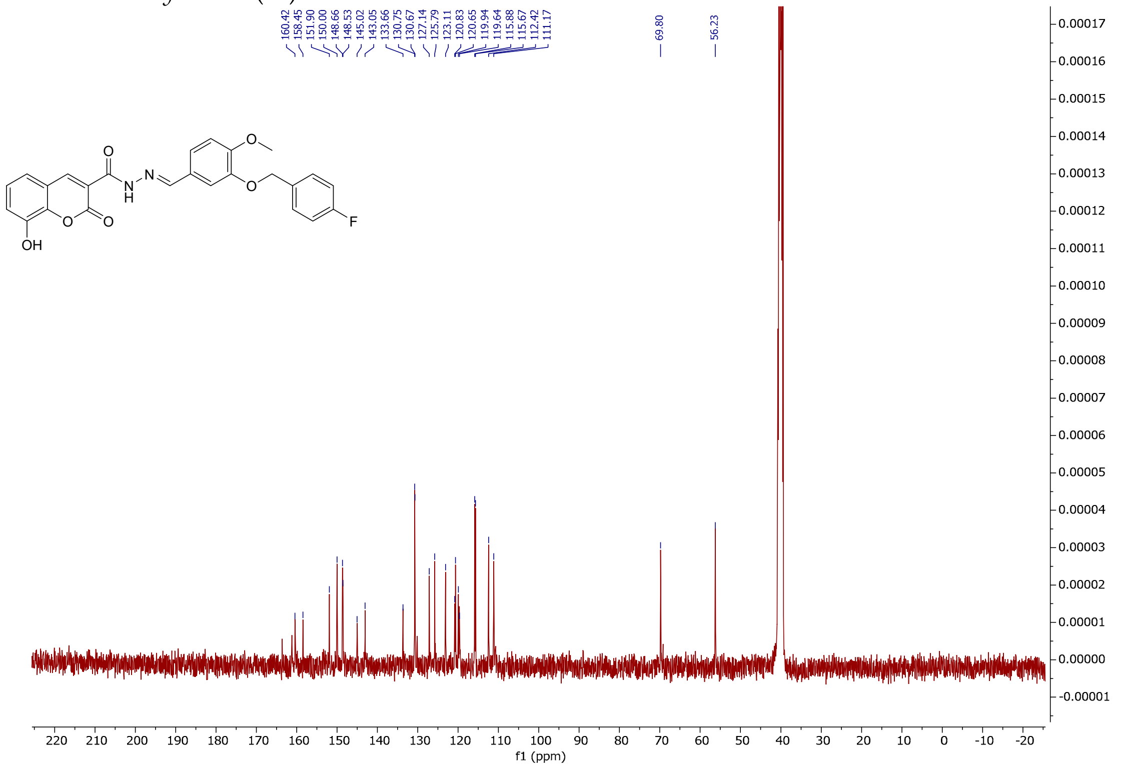

Figure S33. [13]H NMR spectra of *(E)-N′-(3,4-dihydroxybenzylidene)-8-hydroxy-2-oxo-2H-chromene-3-carbohydrazide* (**17**).

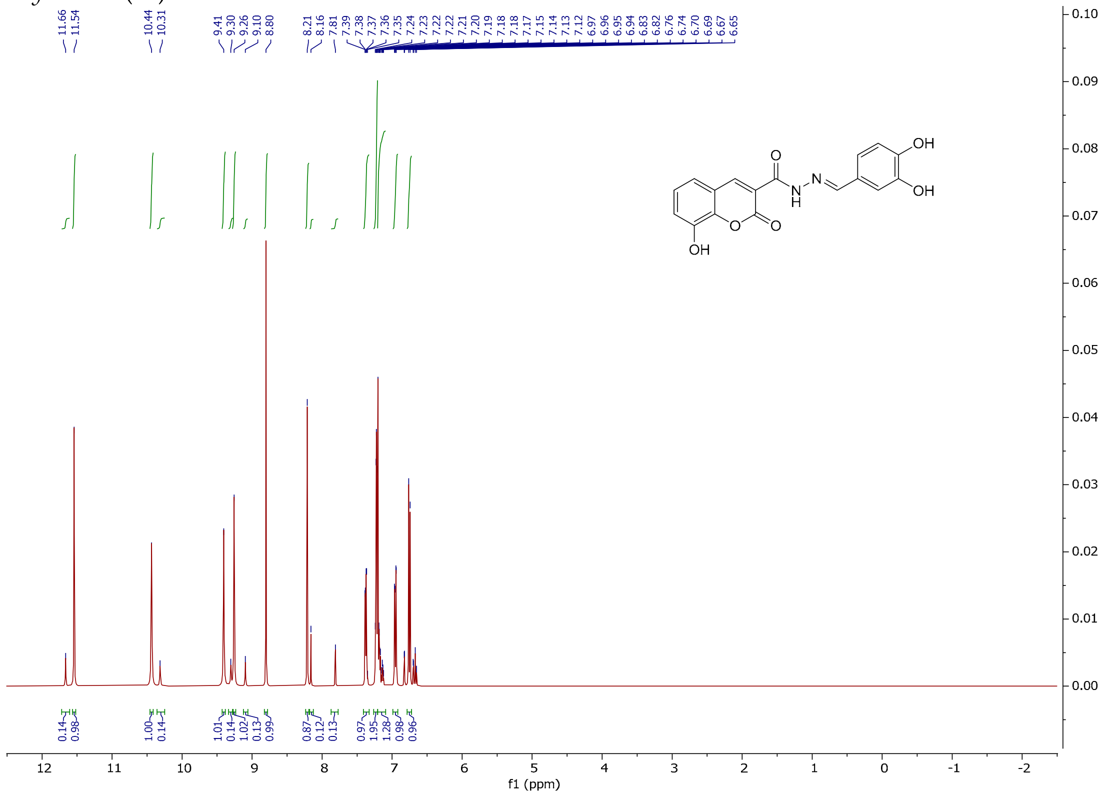

Figure S34. ^{13}C NMR spectra of *(E)-N′-(3,4-dihydroxybenzylidene)-8-hydroxy-2-oxo-2H-chromene-3-carbohydrazide* (**17**).

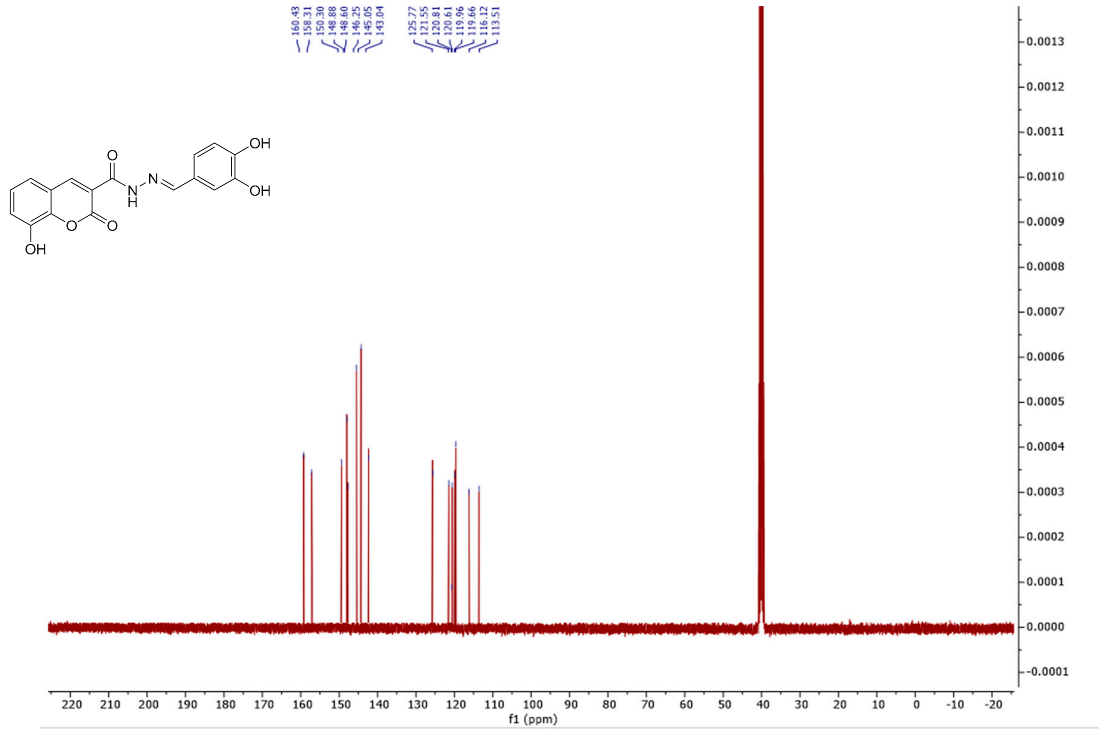

Supplement: Supplementary file 1 [file antioxidants-15-00031-s001.zip › Supporting Materials_16Ott_CG.pdf]
